# How impact factors, including the COVID-19 pandemic, change the quality of life of the elderly

**DOI:** 10.1186/s12963-025-00395-9

**Published:** 2025-07-01

**Authors:** Shuang Cang, Yi Lu

**Affiliations:** 1 Department of Mathematics, College of Engineering, Design and Physical Sciences, Brunel University of London, Uxbridge, UB8 3PH UK; 2 Guangdong Provincial/Zhuhai Key Laboratory of IRADS, Department of Statistics and Data Science, Beijing Normal-Hong Kong Baptist University, Zhuhai, 519087 China

**Keywords:** Quality of life, Psychological distress, Activity, Companionship, Medical conditions

## Abstract

**Purpose:**

The worldwide population is facing the aging issue. Additionally, the COVID-19 pandemic has decreased the living standards of elders. Therefore, understanding the impact factors changing the quality of life of the elderly, and considering the effects of the COVID-19 pandemic, has become a focus for central and local governments, as well as individual families.

**Methods:**

This study analyses newly designed comprehensive relationship networks related to the quality of life of elders in association with the COVID-19 phenomenon. Construct validity was assessed using exploratory factor analysis and confirmatory factor analysis. A partial least squares structural equation model was employed to identify the path relationships. Multiple logistic regression was conducted to investigate the impact of demographic information on the quality of life of the elderly.

**Results:**

The findings indicate that the quality of life is directly and strongly influenced by factors such as psychological distress, COVID-19, quality of daily living, and group and individual activities. Companionship particularly affects the latter two factors for elderly individuals. Additionally, COVID-19 significantly impacts companionship due to the perceived isolation it brings to the elderly. Furthermore, the medical condition factor affects psychological distress, suggesting that the health status of the elderly influences their mental well-being. Moreover, the good diet quality factor strongly influences the spiritual and material aspects of life as well as the mental and physical health of elders. Other factors influencing the physical health of elders include demographics, gender, age, marital status, and homeownership status.

**Conclusion:**

These findings show the necessity of taking care of, accompanying, and improving the medication conditions of the elderly, especially during the period of COVID-19.

## Introduction

Population aging is expected to have a profound impact on the world. The World Population Prospects predicts that the life expectancy at birth will rise from 71 years in 2010–2015 to 77 years in 2045–2050 [40]. The population aged 60 and over is growing faster at a rate of about 3% per year globally. In Asia, the population is expected to increase from being 12% of the total in 2017 to 24% in 2050 [40]. Aging induced changes, such as physical or cognitive decline, may affect the elders’ quality of life (QoL), leading to injury, mental health issues, or physical inactivity.

The outbreak of the COVID-19 pandemic puts a strain on people’s daily lives, especially for older individuals. The elderly is more likely to be infected by COVID-19 and exposed to the danger of death as a result. Additionally, elderly people have experienced home isolation or reduced contact with their relatives and friends, being unable to participate in group activities due to COVID-19 [26, 37]. How to maintain or improve the QoL of the elderly in this severe situation is a great challenge.

The World Health Organization (WHO) defined QoL as “individuals’ perception of their position in life in the context of the culture and value systems in which they live and in relation to their goals, expectations, standards and concerns [12]”. Building upon this definition, this paper examines how the QoL of the elderly will be affected by their daily lives, especially under the COVID-19 pandemic situation. Existing studies mainly discuss the impact of individual factors on the QoL of the elderly, lacking a comprehensive approach that considers physical, mental, experiential, and COVID-19 related elements together. This study will fill in the gap by analysing these multiple factors and collecting the latest data to help elders cope with the previously mentioned changes and challenges brought about by the COVID-19 pandemic.

The aim of this study is to propose a comprehensive conceptual model to analyse the relationship among the factors influencing the QoL for the elderly under the COVID-19 pandemic situation. This analysis will help improve elders’ daily lives and health and provide guidance for local and central governments in policymaking.

## Literature review and new conceptual model

A significant amount of literature has investigated the various influences on the QoL of the elderly. Researchers concentrate more on individual aspects, such as daily activity, physical health, psychological health including mental health problems and stress, and social relationships such as connections with friends and relatives. Because COVID-19 has a significant impact on the QoL of the elderly, factors related to the pandemic COVID-19 need to be measured [24]. Colucci et al. [8] found that the physical activity directly affects the QoL of the elderly. Appropriate physical activities are necessary and helpful in maintaining or restoring the health of the elderly. Apart from physical activities, daily life behaviours such as sleep quality have direct impacts on both the health and QoL of the elderly [45, 48].

This study considers the following research question: What are the main factors influencing the QoL of the elderly under the COVID-19 situation? The hypotheses are listed below:

### H1

COVID-19 related effects.

### H2

Psychological distress.

### H3

Quality of daily life.

### H4

Group activity.

### H5

Individual activity.

As they age, most elderly individuals develop chronic diseases such as hypertension, hyperlipidemia and hyperglycemia. They may also suffer from multiple diseases simultaneously, meaning two or more chronic diseases at the same time [26, 42]. COVID-19 mortality is also connected with comorbidity [39]. Yang et al. [42] pointed out that chronic diseases increase the psychological pressure on the elderly. Li and Shou [23] found that such depressive symptoms can be severe, affecting the QoL of older patients, and may even lead to suicide in serious cases. Li and Shou [23] suggested that ‘as the gatekeepers of resident health, GPs should pay more attention to elderly mental and psychological disorders while treating their physical illnesses.’ Therefore, the hypotheses below are designed.

### H6

The effects of medical conditions on the COVID-19 related situation.

### H7

The effects of medical conditions on psychological distress.

### H8

The effects of medical conditions on group activity.

Social supports such as family care, friend’s support, or community care are all external supports for the elderly and can help them physically and psychologically [26, 42]. Relationships such marital status indirectly affect the depressive symptoms of the elderly by mediating with daily pattern and life satisfaction [32]. The intergenerational relationship can improve elders’ depression level while family support can also be a mediation. In this study, the factor of companionship is added, this factor concentrates on the elderly’s subjective feelings of companionship in addition to the physical social companionship. The social support moderated the negative effects of chronic diseases on psychological pain to some extent, and enhanced the positive mental health of the elderly.

The research questions are: (1) Does companionship promote elderly’s individual and group activities? (2) Does companionship affect the daily life quality of the elderly? The associated hypotheses are listed below:

### H9

Companionship’s effects on psychological distress.

### H10

Companionship’s effects on quality of daily life.

### H11

Companionship’s effects on group activity.

### H12

Companionship’s effects on individual activity.

Psychological diseases such as depression are one of the main causes of emotional distress in the elderly, and they are related to their daily agenda, such as diet quality [9]. Physical abuse by Chinese parents in childhood is related to mental distress, and may lead to depression in middle-aged and older groups [49]. A beneficial childhood experience can indirectly promote longevity and health in old age by improving socioeconomic status in adulthood [36]. Group activities such as singing and dancing are perceived as beneficial to their well-being and mental health [5, 21, 34], but whether individual activities can relieve psychological distress will be tested through the following hypothesis:

### H13

Individual activities have an impact on psychological distress.

The outbreak of COVID-19 has affected almost every aspect of people’s lives around the world, especially the health of the elderly. Since the elderly are more vulnerable to diseases, they need more protection from the pandemic [8]. As one of the protective measures, the lockdowns are thought to have an indirect impact on the QoL of the elderly. Colucci et al. [8] found that a decrease in QoL, perceived health and well-being were observed between T1 (December 2019 before Covid-19) and T2 (June 2020 first lockdown) and between T1 and T3 (January 2021 s lockdown). The COVID-19 pandemic also increases the risk of subjective memory decline [14], and is relates to anxiety and wellbeing [27]. COVID-19’s effect on individual activity has been studied [31], thus the hypotheses are as below:

### H14

COVID-19’s effect on companionship.

### H15

COVID-19’s effect on group activity.

Apart from the main factors mentioned above, there are many other factors according to different applications and problems. Yu et al. [44] examined the building environments and care services of rural nursing homes in their case study. They found that the QoL of the elderly could be accurately predicted from the building environment, such as room distance and space, as well as daily care services. The relationship between parental influences and the health of the elderly has been studied by Liu et al. [25] who found that parental influences have a positive impact on the health of male children and a negative impact on female children when they become elderly.

**Fig. 1:**
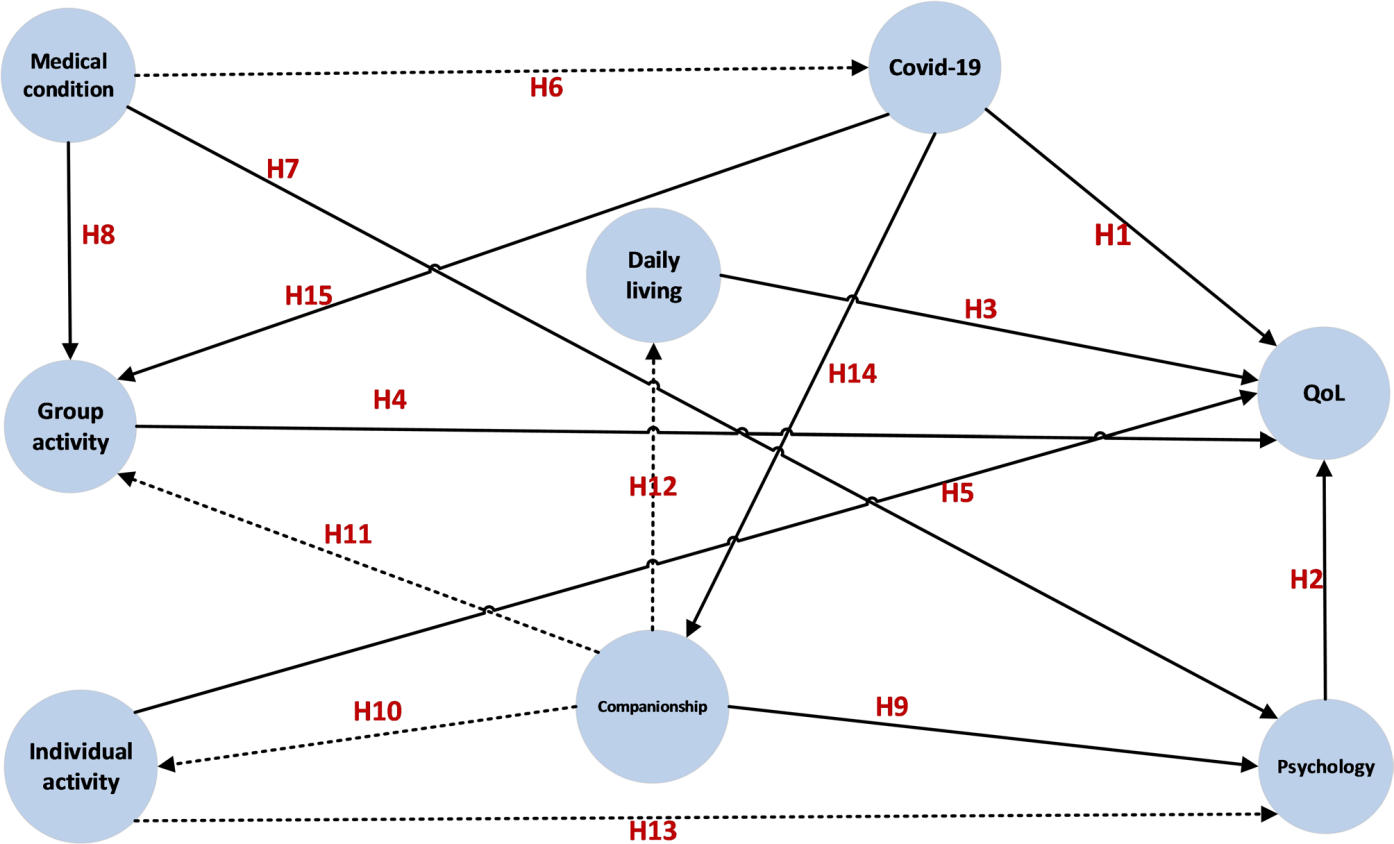
The proposed conceptual model. Note: Solid line: the researched paths in the existing literature; Dot line: the newly designed research paths in this study

The COVID-19 related situations are investigated in this study and some existing conclusions are reassessed in the network. The highly considered question is 3) Does the COVID-19 related situations directly effect on the QoL for the elderly? For the isolation related by COVID-19 pandemic, it may influence the companionship. Therefore, the research questions are 4) Does the COVID-19 related situations impact on the companionship of the elderly? 5) How does the medical condition of the elderly relate with Covid-19? Base on the research questions and hypotheses listed before, a new conceptual model is proposed in Fig. 1. There are 15 paths in this model and 8 factors connected by these paths.

### Methods

### Primary data and questionnaire design

In this study, primary data was collected in person at Zhuhai city, Guangdong province through questionnaires. Zhuhai has a unique geographical location, bordering Macao and Hong Kong. The city is surrounded by the sea with pleasant weather, green plants, and flowers almost everywhere. The government focuses on the development of elderly care such as elderly canteens and activity centres. It is almost the first choice and ideal retirement home for the elderly people in mainland China, Hong Kong and Macau. Before data collection, the sample size is calculated using the formula $\:{Z}_{\alpha\:/2}^{2}\times\:p\times\:(1-p)/{E}^{2}$. *Z* refers to the test statistic value, which is set to 1.96, corresponding to the 95% confidence level with two tails. The value *p* is the probability of the population while *E* denotes the desired margin of error, which is set to the standard value of 0.05. Thus, a sample size of 385 is needed. The data was collected from May to July, 2022, with the help of social workers and volunteers. This study utilized a multistage sampling method, where clusters were randomly selected, and then samples within the selected clusters were randomly chosen. It started with the districts of Zhuhai, which were randomly selected, followed by communities within the towns/streets of the selected districts. Finally, the sample size for each community was determined, and samples were randomly selected from each of these 11 designated communities.

About 550 questionnaires were distributed in these communities in Zhuhai for elderly people aged 60 and above, because the official latest retire age is 60 in China. After screening out the invalid questionnaires such as those with missing values, 410 questionnaires are selected to be analysed.

The detailed questionnaire items are divided into three parts: demographic and characteristic information (see Table 1), and the five-point scale for the eight factors, as shown in Table 2. These factors, using a five-point scale, were designed to range in the same direction. For the factors ‘Quality of Life’, ‘Quality of Daily Living’, and ‘COVID-19-related situation’, the scale is: 1 = Very bad, 2 = Bad, 3 = Average, 4 = Good, and 5 = Very good. For the factors ‘Psychological Distress’ and ‘Companionship’, the scale is: 1 = Never, 2 = Rarely, 3 = Sometimes, 4 = Often, and 5 = Always. For the factor ‘Medical Condition’, the scale is: 1 = Very inconsistent, 2 = Inconsistent, 3 = Neutral, 4 = Consistent, and 5 = Very consistent. For the factors ‘Group Activity’ and ‘Individual Activity’, the scale is: 1 = not participate, 2 = 1–2 times, 3 = 3–4 times, 4 = 5–6 times, and 5 = 7 times or more.

The partial least square structural equation model (PLS-SEM) is used to analyse the relationships among these eight factors, as referenced in the conceptual model (Fig. 1). Multiple logistic regression is used to study the impact of demographic and characteristic information, as well as the other seven factors (excluding the QoL factor), on the QoL, which consists of four items: ‘Enriched in my spiritual life’, ‘Enriched in my material life’, ‘Good mental health’, and ‘Good physical health’.

### Construct of the conceptual model

The conceptual model comprises eight factors that are related to the QoL of the elderly. The first factor, QoL, a dependent variable, covers five items: ‘happy most of the time’, ‘abundance in spirit’, ‘abundance in material life,’ ‘good physical health’ and ‘good mental health.’

The second factor ‘Psychological distress’ considers the following aspects: Depression is one of the main causes of emotional distress in the elderly, which has a great impact on their life and health [32]. Additionally, elderly people may easily experience psychological stress due to illness [42]. Moreover, childhood illnesses or trauma brought about by threatening violence may also affect one’s later life. Furthermore, if parents suffer from mental illness, children may become vulnerable to mental stress, which may hinder the growth of children. Mental and economic stress are also considered in the research.

The third factor ‘Medical condition’ is also a very important factor influencing the QoL of the elderly. Elders may suffer from pain due to gradual weakness and diseases and long-lasting pain could interfere with their daily life [32]. Due to illness and the aging condition, their energy tends to become lower each year, and they may easily run out of energy [8]. Taking medicine regularly is key in preventing chronic diseases.

The fourth factor ‘Quality of daily living’ includes six items: sleep, diet, surrounding environment, relationship with children, childhood health and childhood happiness. Most elders have sleep problems due to diseases or other reasons, thus, sleep quality is an important factor to consider. Diet reflects whether the elderly have a balanced food consumption, which mediates sleep quality and health [9, 48]. Because most of the elderly spend more time at home, especially during the COVID-19 pandemic period, the living environment is also an important aspect. Many articles have found that the community and neighbourhood environment influence elderly’s psychological condition [22, 46, 47]. In China, the elders’ relationship with their children will also make a difference on their QoL. This can be described as the intergenerational relationship, which refers to the interpersonal relationship between two generations such as the elderly parents and their adult children. This relationship is often of a great concern to the elderly and is an integral part of their lives. In addition to the intergenerational relationship with their children, Shen and Zeng [36] and Zheng et al. [49] stated that the elders’ own childhood situations may also affect their QoL when they get old.

The fifth factor ‘Group activity’ factor considers leisure travel, square dance, cultural activities, social activities (meeting and connecting with friends, family and community members) and other collective activities such as club gatherings. Leisure travel activities such as visiting distant relatives and friends and getting in touch with nature are common for the elderly, and these can positively improve their lives [2, 33]. Such activities also improve their psychological health [38, 51]. In recent years, square dance has become a popular and essential activity for elders in China [50]. Many older people also take part in cultural activities such as singing and playing chess with their friends. In Chinese culture, people build deep connections with their relatives, thus the elderly usually participate in family events such as family dinner or gatherings.

The sixth factor is related to COVID-19, covering four items: life changes caused by the pandemic, willingness to take the vaccination, social relations, and self-rated energy level [4]. Specifically, how people react to social distance measures, the nucleic acid testing, and lockdown will be studied. Vaccination can effectively prevent infection during the pandemic and the willingness to take vaccination also makes differences [4, 7]. However, some elders may not be willing to take it due to old age and illness conditions. People’s resilience and psychological flexibility in coping with the pandemic vary, which will also influence their lives differently [27, 29, 35].

The seventh factor is Companionship. Elders often experience feelings of loneliness and a desire for companionship with relatives or friends. Khalaila and Vitman‑Schorr [20] studied that companionship might reduce the QoL of care-recipients by increasing their feelings of loneliness, social isolation and reduced perceived family support. However, there is a limit to how long their family members or friends can stay with them [17, 26]. This is because they may be occupied with work or their own family lives. This study considers this phenomenon and incorporates the companionship factor into the conceptual model.

The last factor focuses on Individual activity. Taking the age and energy level of elders into consideration, we examine the types of independent physical activities they are involved in, as well as the duration and intensity level of these activities [42]. With the rapid development of social media, elders can now participate in social activities on their own without face-to-face interactions through social software such as WeChat and Facebook. They can also use smartphones to read news and shop online at home, especially during the COVID-19 pandemic period.

### Statistical methods

This study uses the exploratory factor analysis (EFA) method to find the underlying impact factors. and the structural equation model (SEM) to identify the impact relationship among these multiple factors, this approach is more powerful than multiple regression analysis, which examines only many-to-one relationships. The advantages of using SEM are (1) validity; (2) reliability; (3) confirmatory approach. To identify the impact factor relationships, this study uses PLS-SEM [18] together with bootstrapping 50,000 subsample. The bootstrapping method can be used to evaluate the overall model fitting in PLS [10].

## Results

### Demographic information

Table 1 shows that the average age of the participants is 68.77 years with the standard deviation of 6.765. The percentage of age distribution is 59.51% for ages 60–69, 30% for ages 70–79 and 10.48% for age 80 and above. It roughly matches the age composition percentage of Chinese people at the national level, which are 60–69 (55.82%), 70–79 (30.63%), 80 and above (13.55%) (National Bureau of Statistics, 2021). According to the China statistical yearbook (2021), the percentage of male is less than female for the 60 + age group. Since the samples of this study were collected in outdoor settings, many of the participants were female who are more likely to attend outdoor activities. Regarding marital status, most of the elderly are married (75.21%) from the National Bureau of Statistics, which is close to 81.2% of the sample studied.

**Table 1:** Demographic & characteristic information of the response

Sample size: 410; Age Average: 68.77; Age Standard deviation: 6.765					
**Age**	**N**	**Per.%**	**Gender**	**N**	**Per. %**
60–69	244	59.50	Male	112	27.30
70–79	123	30.00	Female	296	72.20
80–89	43	10.50	Missing	2	0.5
**Education level**	**N**	**Per. %**	**Marital status**	**N**	**Per. %**
Primary school or below	58	14.1	Unmarried	2	0.5
Junior high school	91	22.2	Death of a spouse	50	12.2
High/technical school	138	33.7	Divorced	13	3.2
University or junior college	113	27.6	Married	333	81.2
Master degree or above	5	1.2	Missing	12	2.9
Missing	5	1.2	**Household member**	**N**	**Per. %**
**Number of children**	**N**	**Per. %**	Living alone	32	7.8
0	3	0.7	Living with husband/wife	176	42.9
1	186	45.4	Living with children	193	47.1
2	125	30.5	Living with other family	6	1.5
>=3	72	17.6	Missing	3	0.7
missing	24	5.9	**House ownership type**	**N**	**Per. %**
**Residence status**	**N**	**Per. %**	Individual ownership	208	50.7
No	85	20.7	Family ownership	163	39.8
Yes	317	77.3	Rent	25	6.1
Missing	8	2.0	Missing	14	3.4

N = number; Per.=percentage

### Construct underlying factor and reliability

The Construct validity is analysed by exploratory factor analysis (EFA) and confirmatory factor analysis (CFA) [11, 28], EFA used principal component analysis and Varimax rotation to extract factors, and CFA tested the original four-factor model. The Exploratory factor analysis (EFA) is performed on the SPSS version 25 software. The initial 38 items became 35 items because three items with loadings less than 0.4 are deleted [16]. One such item is ‘constantly worry about Covid-19’. Additionally, although nine factors are initially identified with eigenvalues greater than or equal to one, only eight factors are used to conduct the EFA, as one factor had only one item.

The Kaiser-Meyer-Olkin (KMO) statistic for all the items is 0.855, and the Bartlett’s test of sphericity shows it highly significant at *p* < 0.001 (Chi-Square = 6527.001), indicating that the data is suitable to be analysed by EFA [19]. The result of EFA using the 35 items is shown in Table 2.

**Table 2:** Results of EFA

Factors and items	Factor loading	Eig.	VE (%)	AVE (%)	$\:\alpha\:$	Mean
**Factor 1: Quality of life**		4.335	12.386	12.386	0.915	3.778
Happy most of the time	0.739					
Abundance in spirit	0.801					
Abundance in material life	0.790					
Good physical health	0.706					
Good mental health	0.807					
**Factor 2: Psychological distress**		3.130	8.943	21.328	0.827	3.614
Feel down in the dumps	0.763					
Have mental stress	0.790					
Have economic stress	0.750					
Your parents often feel uneasy and anxious	0.668					
Beaten/shouted by parents in childhood	0.686					
**Factor 3: Medical condition**		2.875	8.214	29.543	0.825	3.284
Constant physical pain	0.792					
Physical problems caused by chronic disease (s)	0.888					
Mental problems caused by chronic disease (s)	0.799					
Difficult to take medicine regularly	0.568					
**Factor 4: Quality of daily living**		2.745	7.843	37.386	0.792	3.575
Good sleep quality	0.511					
Good diet quality (balanced nutrition)	0.561					
Good surrounding environment	0.703					
Good relationship with children if one has them	0.716					
Healthier childhood	0.627					
Happy childhood	0.463					
**Factor 5: Group activity** **(Expect COVID-19 lockdown)**		2.406	6.875	44.261	0.624	2.137
Leisure travel activities (measured as “stay more than one night”)	0.510					
Square dance	0.620					
Cultural activities such as singing	0.586					
Meeting and connecting with friends, family and community members	0.508					
Other group activities such as club, religion activities	0.769					
**Factor 6: Covid-19 related situation**		2.141	6.117	50.379	0.673	3.674
Willingness to adapt to changes (nucleic acids, lockdown etc.)	0.720					
Willingness to be vaccinated	0.704					
Normal social relations such as relationship with friends	0.492					
Good energy (does not get tired easily)	0.414					
**Factor 7: Companionship**		2.048	5.851	56.230	0.692	3.576
Accompanied by relatives	0.786					
Accompanied by friends	0.735					
Feeling being valuable (helpful) to your relatives/friends	0.485					
**Factor 8: Individual activity**		1.565	4.472	60.702	0.642	2.472
Using social software such as WeChat	0.590					
Using phone and shop online	0.680					
Medium/high physical activities such as mountain climbing	0.679					

Note: Extraction Method: Principal Component Analysis. Rotation Method: Varimax with Kaiser Normalization, rotation converged in 19 iterations

Eig.=Eigenvalue; VE = Variance explained; AVE = Accumulated variance explained

$$\:\alpha\:$$: Cronbach’s alpha

Table 2 displays eight factors, with the factor ‘Quality of life’ having the highest percentage of explained variance, followed by ‘Psychological distress’, ‘Quality of daily living’, ‘Medical conditions’, ‘Group activities’, ‘COVID-19 related’, ‘Companionship’ and ‘Individual activities.’ The total accumulated variance explained is over 60%, indicating that more than 60% of the information is extracted by these eight factors. The Cronbach’s alpha values for factors 1 to 4 exceed 0.7, while those for factors 5 and 8 are close to 0.7, thus confirming the validity of the dataset.

## Relationship identification

**Fig. 2:**
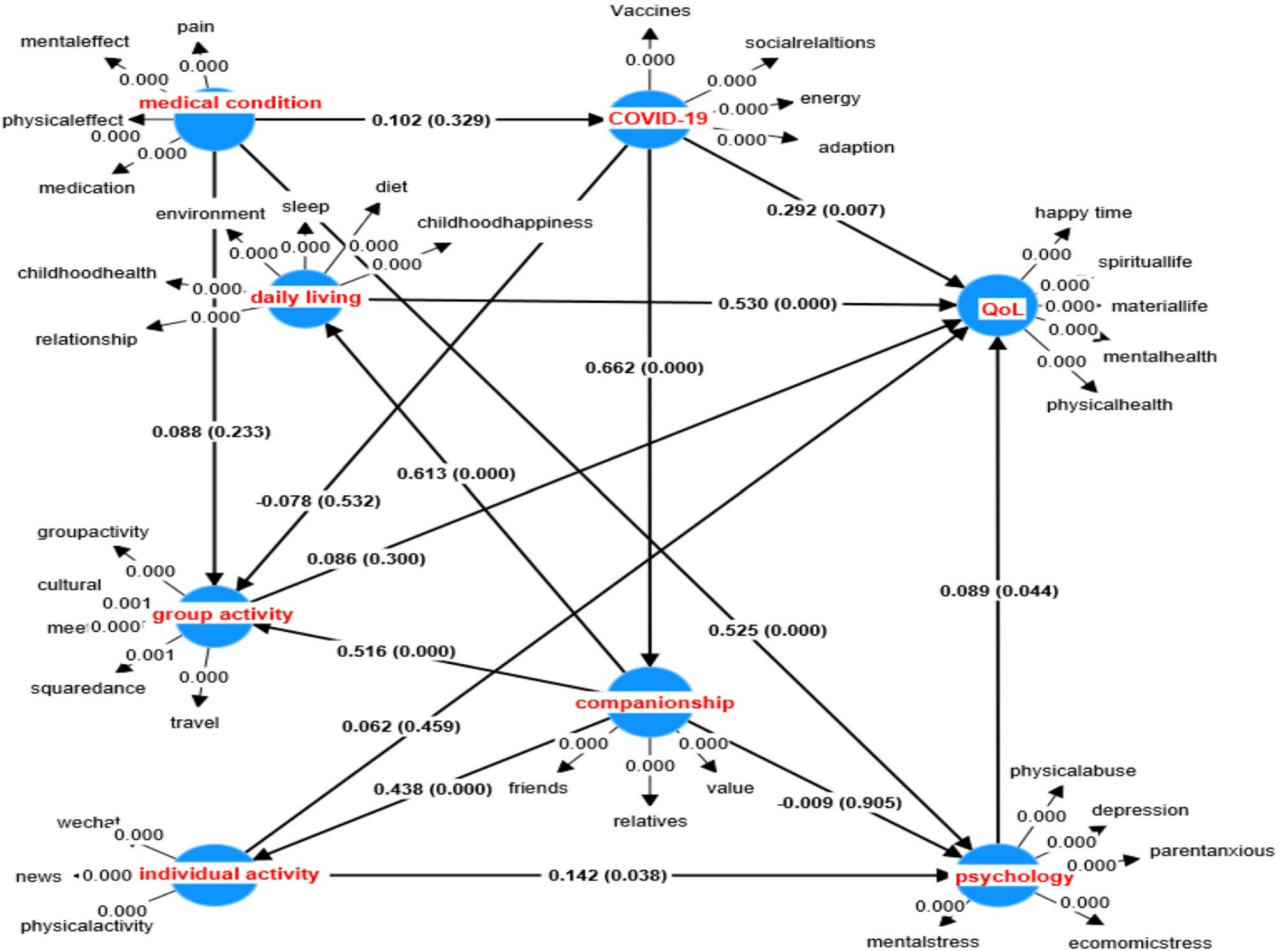
Outputs from PLS-SEM bootstrapping

PLS-SEM with bootstrapping is used to analyse these 35 items with 8 factors. The analysis results including the path coefficients and the P-values are shown in Fig. 2. If the P-value associated with a path coefficient is less than 0.01, the impact is considered strongly significant. If the P-value is greater than 0.01, but less than 0.05, the impact is still considered as significant, but less strong.

The outcomes of significant path analysis from PLS-SEM are illustrated in Table 3.

**Table 3:** Path effects using consistent PLS-SEM bootstrapping (subsamples: 50000)

	Indirect Effect		Direct Effect		Hypothesis
			T	*P* value	
**H1**: Covid-19-> QoL	4.783	0**	2.699	0.007**	Strong support
**H14**: Covid-19-> companionship			10.401	0**	Strong support
**H15**: Covid-19-> group activity	0.826	0.409	0.625	0.532	No support
**H2**: psychology -> QoL			2.017	0.044*	Support
**H7**: companionship -> psychology	1.885	0.059	0.119	0.905	No support
**H8**: companionship -> daily living			8.47	0**	Strong support
**H9**: companionship -> group activity			4.474	0**	Strong support
**H10**: companionship-> individual activity			6.741	0**	Strong support
**H3**: daily living -> QoL			5.334	0**	Strong support
**H5**: group activity -> QoL			1.036	0.3	No support
**H6**: individual activity -> psychology			2.075	0.038*	Support
**H4**: individual activity -> QoL	1.451	0.147	0.74	0.459	No support
**H11**: medical condition -> Covid-19			0.977	0.329	No support
**H12**: medical condition -> psychology	0.424	0.671	8.993	0**	Strong support
**H13**: medical condition -> group activity	0.837	0.403	1.193	0.233	No support

**: *P* < 0.01; *: $$\:0.01\le\:$$*P*<0.05

The results from Table 3 are outlined below:

Factor 6 (COVID-19) has a strongly significant impact on the QoL of elders, supporting existing literature that highlights the influence of COVID-19 related situations on QoL of the elderly [26, 36, 45]. Additionally, COVID-19 has a strongly significant impact on Factor 7 (Companionship), which strongly supports that COVID-19 related situations increase the level of perceived isolation [8]. However, the path from Factor 6 (COVID-19) to Factor 5 (Group activity) is not significantly related.

Factor 2 (psychological distress) and Factor 4 (quality of daily living) directly affect Factor 1 (QoL) at the significant levels of 1% and 5%, respectively, this suggests that the quality of life of the elderly is related to their daily activities and psychological conditions [36, 45], emphasizing the strong influence of daily life on the QoL of the elderly [43, 48]. However, Factor 5 (group activity) and Factor 8 (individual activity) show no impact on the QoL of the elderly, while Factor 8 (individual activity) demonstrates a significant impact on Factor 2 (psychological distress).

Factor 3 (medical condition) has a strongly significant impact on Factor 2 (psychological distress) at a significance level of 1%, illustrating that the medical conditions of the elderly strongly influence their mental health. This result goes in line with the conclusion drawn by Yang and D’Arcy [42]. But Factor 3 (medical condition) has no impact on Factor 5 (group activity) and Factor 6 (COVID-19 related situations) in this study, indicating that in general the elderly can adapt to the changing situations related to COVID-19 and participate in group activities as usual regardless of their medical conditions.

Factor 7 (companionship) has a strongly significant impact on Factor 4 (quality of daily living), Factor 5 (group activity) and Factor 8 (individual activity) at the significant level of 1%, which shows the importance of companionship for the elderly. However, Factor 7 (companionship) does not have an impact on Factor 2 (psychological distress). This differs from Li and Shou’s [23] study, which suggests that psychological distress can be improved by companionship and physical activities [14, 15, 37]. This may be caused by the restriction during COVID-19 pandemic.

Table 3 shows a strongly significant indirect effect of the COVID-19 pandemic on QoL, daily living, group and individual activities of the elderly. Additionally, it reveals a strongly significant impact of companionship on the QoL of the elderly.

Tables 3 and 4 indicate that the ‘Covid-19-> group activity’ path is fully medicated, while the ‘Covid-19-> QoL’ path is partially medicated. ‘Full medication’ is defined as the direct effect being non-significant, while the indirect effect is significant; ‘partial medication’ means both the direct and indirect effects are significant.

Overall, the paths involving the companionship factor are significant, indicating that companionship plays an essential role in the relationship network. Companionship significantly impacts the quality of daily living, group and individual activities at a significant level of 1%. Conversely, the COVID-19 pandemic influences companionship, likely due to the distant measure and the quarantine policies. It is noteworthy that medical conditions strongly influence psychological distress. Furthermore, factors such as psychological distress, daily living, and COVID-19 have a direct effect on the QoL of elders. These findings are consistent with prior research indicating the effects of medical conditions on psychological distress [42], psychological distress and quality of daily living [36, 45], and direct effects of COVID-19 on the QoL of the QoL of the elderly [27, 35]. In summary, to enhance the QoL of the elderly, attention should be paid to their psychological well-being and daily living conditions.

### Impact factors on QoL of the elderly

It is important to identify the factors impacting the QoL of elders. In this study, four items are used as dependent variables, comprising questions related to the spiritual and material aspects of life, as well as mental and physical health. The average values of each of these seven factors listed in Table 4 are used as input variables, and the demographic and characteristic information is used as input to conduct multiple logistic regression separately. The results are shown in Table 4. The deviance values from the goodness of fit are not significant at the 5% level, indicating a good fit of the model to the data.

**Table 4:** Impact on QoL analysis using multiple logistic regression

Q1: Enriched in my spiritual life; Q2: Enriched in my material life; Q3: Good mental health; Q4: Good physical health				
**Factor QoL**	**Q1**	**Q2**	**Q3**	**Q4**
**Factor 2: Psychological distress**	0.008**	0.011*	**0.072**	**0.092**
Feel down in the dumps	0.474	0.866	0.136	0.982
Have mental stress	0.109	0.863	0.475	0.041*
Have economic stress	0.867	0.015*	0.738	0.013*
Your parents often feel uneasy and anxious	0.934	0.794	0.567	0.801
Beaten & shouted by parents in childhood	0.470	0.785	0.697	0.050
**Factor 3: Medical condition**	**0.933**	**0.417**	**0.630**	0.000**
Constant physical pain	0.567	0.975	0.891	0.092
Chronic disease causes physical problem	0.374	0.125	0.850	0.021*
Chronic disease causes mental problem	0.002**	0.000**	0.103	0.556
Difficult to take medicine regularly	0.723	0.431	0.754	0.149
**Factor 4: Quality of daily living**	0.000**	0.000**	0.000**	0.000**
Good sleep quality	0.139	0.704	0.048*	0.001**
Good diet quality (balanced nutrition)	0.000**	0.000**	0.000**	0.000**
Good surrounding environment quality	0.413	0.237	0.474	0.254
Good relationship with children if have	0.000**	0.048*	0.050	0.500
Healthier childhood	0.110	0.463	0.607	0.156
Happy childhood	0.000**	0.000**	0.064	0.086
**Factor 5: Group activity** **(Except COVID-19 lockdown)**	**0.892**	**0.256**	**0.541**	0.011*
Leisure travel activities (measure as “stay more than one night”)	0.025*	0.142	0.168	0.086
Square dance	0.434	0.415	0.687	0.342
Cultural activities such as singing	0.437	0.570	0.957	0.162
Meeting and connecting with friends, family and community members	0.176	0.081	0.124	0.400
Other group activities such as club activities	0.237	0.105	0.094	0.426
**Factor 6: COVID-19 related situations (During COVD-19 pandemic period)**	0.000**	0.000**	0.001**	0.000**
Willing to adapt changes (nucleic acids/lockdown etc.)	0.114	0.294	0.283	0.200
Willing to be vaccinated	0.005**	0.034*	0.044*	0.114
Normal social relations such as the relationship with friends	0.000**	0.000**	0.000**	0.000**
Good energy (doesn’t get tired easily)	0.003**	0.001**	0.012*	0.000**
**Factor 7: Companionship (Often)**	0.006**	**0.155**	**0.206**	**0.497**
Accompanied by relatives	0.351	0.749	0.779	0.040*
Accompanied by friends	0.003**	0.013*	0.009**	0.000**
Valuable (helpful) to your relatives/friends	0.000**	0.000**	0.000**	0.001**
**Factor 8: Individual activity**	**0.139**	**0.279**	**0.458**	**0.725**
Using social software such as WeChat	0.032*	0.242	0.127	0.285
Using phone and shopping online	0.348	0.442	0.867	0.918
Medium/high physical activities such as mountain climbing	0.042*	0.101	0.311	0.011*
**Demographic & Characteristic information**	**Q1**	**Q2**	**Q3**	**Q4**
Age	0.280	0.564	0.939	0.009**
Gender	0.095	0.031*	0.008**	0.202
Education level	0.022*	0.062	0.279	0.639
Number of children	0.748	0.848	0.352	0.939
Marital status	0.499	0.513	0.039*	0.018*
Residence	0.489	0.323	0.011*	0.155
Household member	0.097	0.650	0.078	0.739
House ownership type	0.213	0.151	0.010*	0.002**

**: *P* < 0.01; *: $$\:0.01\le\:$$*P*<0.05

Table 4 illustrates each factor and its associated items’ specific impacts on the QoL of the elderly. Factors 4 (Quality of daily living) and 6 (COVID-19 related situations) have strongly significant impacts on all four items of QoL (Q1, Q2, Q3 and Q4), indicating the important relationship between the COVID-19 pandemic and quality of daily living with the QoL of the elderly. These findings align with those showing that the COVID-19 pandemic impacted the QoL of older adults by fostering social isolation and physical inactivity [6]. For Factor 4, the item ‘good diet quality (balanced nutrition)’ has a strongly significant impact on all the dependent variables, highlighting the importance of balanced nutrition. Poor nutritional status among COVID-19 patients was associated with adverse health outcomes and impaired QOL was found by Shikieri et al. in Saudi Arabia [13]. Additionally, the items ‘happy childhood’ and ‘good relationship with children if have’ of Factor 4 also exhibit a strongly significant impact on spiritual life (Q1) and material life (Q2), while the ‘good sleep quality’ item of Factor 4 significantly influences mental and physical health (Q3 and Q4). QoL is influenced by quality of sleep [1]. However, neither the ‘good surrounding environment quality’, nor the ‘healthier childhood’ item has an impact on elders’ QoL, which enhances the existing literature. Regarding Factor 6, ‘normal social relations’ and ‘good energy’ significantly impact on the QoL of the elderly during the COVID-19 pandemic.

Factor 3 (Medical condition) and Factor 5 (Group activity) only significantly affect physical health (Q4), but not so for the variables Q1 to Q3, indicating that group activities and medical conditions can positively changes elders’ physical health. The item ‘chronic disease causes physical problem’ of Factor 3 significantly affects physical health (Q4), while the item ‘chronic disease causes mental problem’ has a strongly significant impact on the spiritual life (Q1) of the elderly. Factor 7 (Companionship) enriches the spiritual life of elders (Q1) and its item ‘accompanied by friends’ significantly impacts all items of the QoL of the elderly, highlighting the importance of companionship once more. Regarding Factor 8 (Individual activity), the items ‘using social software such as WeChat’ and ‘medium/high physical activities’ significantly enrich spiritual life, which is under investigation. The item ‘medium/high physical activities’ also influences the physical health of the elderly. Factor 2 (Psychological distress) influences the spiritual and material life of the elderly. The item ‘have economic stress’ impacts the material life and physical health of the elderly, indicating the necessary of economic support for their QoL. Furthermore, the item ‘have mental stress’ significantly impacts physical health as well, this matches the funding that 7.5% of the total effect of past mental health on physical health is accounted for [30].

In terms of the impact of demographic and characteristic information on QoL, age has a strongly significant impact on elders’ physical health (Q4), which aligns with the common knowledge that people’s physical health tends to decline as they age. Education level significantly influences elders’ spiritual life (Q1), while marital status has a significant impact on both their mental health (Q3) and physical health (Q4). Similar findings show that those who were married at both time points reported better physical health than those who became widowed during the interval (significant primarily for women), and those who had never been married (significant primarily for men) [41]. Additionally, whether the participant is a resident of the city influences their mental health. The types of house ownership (individual ownership, family ownership, or rent) significantly impact the mental and physical health of the elderly. From the perspective of Chinese traditional culture, being a homeowner can guarantee the safety and security of the elderly.

Overall, the specific items (Q1-Q4) that represent Quality of Life (QoL) are used as dependent variables in comparison with the general QoL commonly utilized in the literature. The factors ‘Quality of daily living’ and ‘COVID-19 related situations’ have a strongly significant impact on all four items (Q1-Q4). In particular, ‘Good diet quality’ and ‘Normal social relations, such as relationships with friends,’ play an important role on QoL. Aspects of the factor ‘Companionship’ such as ‘Accompanied by friends’ and ‘Valuable to relatives or friends,’ are also important contributors to QoL.

The number of children and household members do not have any impact on the QoL items (Q1-Q4). However, other demographic and characteristic variables listed in Table 1 have varying degrees of influence on these 4 items (Q1-Q4) of QoL.

## Conclusions

### Relationship paths analysis

The QoL of elders is directly influenced by the factors of ‘psychological distress,’ ‘quality of daily living,’ ‘group activities,’ and ‘COVID-19 related situations.’ This finding offers several directions for improving the QoL of the elderly. The ‘Psychological distress’ factor is directly influenced by ‘Medical condition’, indicating that the underlying health conditions of elders may make them more vulnerable to mental stress, particularly during the COVID-19 period. This is because elders with pre-existing health conditions are more likely to be infected by COVID-19 and may even face mortality as a consequence [3]. ‘Companionship’ is crucial for the elderly, and it can have a significant impact their ‘quality of daily living.’ This is especially true during COVID-19, when social distancing has become necessary. People need to pay more attention to the elderly and help improve their daily living conditions. Encouraging the elderly to participate in ‘group and individual activities’ can be beneficial. However, since COVID-19 related situations can influence companionship, it is essential to pay more attention and explore different and creative ways to promote companionship.

### QoL impact analysis

The ‘Quality of daily living’ and ‘COVID-19 related’ factors strongly affect the QoL of elders, spanning from their spiritual to material life, and from their mental to physical health conditions. In particular, ‘Good diet quality (balanced nutrition)’ plays a crucial role in enhancing the quality of daily living. ‘Medical condition’ and ‘Group activity’ are key factors in improving the physical health of the elderly. This suggests that organizing group activities frequently can be beneficial for enhancing elders’ physical health. ‘Companionship’ can enrich the spiritual life of the elderly, and the items ‘Accompanied by friends and relatives’ influences all items of their QoL, including spiritual, material, mental, and physical health, this demonstrates the vital role of companionship for the elderly. Notably, the item ‘Chronic disease causes physical problem’ affects physical health, while the ‘Chronic disease causes mental problem’ item influences spiritual life. The factor ‘Psychological distress’ also affects elders’ spiritual life, while the item ‘Have economic stress’ influences their material life and physical health. These findings suggest the importance of maintaining or improving the economic condition of elders to alleviate their economic stress.

In terms of demographic and characteristics information, age and marital status significantly impact the physical health of elders, while house ownership type is also crucial for their mental and physical health.

This study contributes to expanding the scope of the literature on the topic. The findings will provide references and data support for the formulation of elderly care policy and the development of the elderly care industry. This evidence can aid in improving the QoL of the elderly and assist governments and organizations in developing new strategies and policies to protect and enhance the QoL of the elderly.

During the COVID-19 pandemic, data collection is challenging. Contacting the elderly requires protective measures and many elders may be unwilling to fill out the questionnaires. Elders with limited mobility or serious illnesses may have little opportunity to leave their homes. For future research, additional impact factors will be incorporated into the conceptual model, and data collection methods can be refined to further explore these relationships.

## Data Availability

No datasets were generated or analysed during the current study.
